# Ca_V_β-subunit dependence of forward and reverse trafficking of Ca_V_1.2 calcium channels

**DOI:** 10.1186/s13041-022-00930-x

**Published:** 2022-05-09

**Authors:** Laurent Ferron, Sydney D. Guderyan, Ethan J. Smith, Gerald W. Zamponi

**Affiliations:** Department of Physiology and Pharmacology, Alberta Children’s Hospital Research Institute, Hotchkiss Brain Institute, Cumming School of Medicine, University of Calgary, Calgary, AB Canada

**Keywords:** Calcium channel, Trafficking, Ca_V_β auxiliary subunits

## Abstract

Auxiliary Ca_V_β subunits interact with the pore forming Ca_V_α_1_ subunit to promote the plasma membrane expression of high voltage-activated calcium channels and to modulate the biophysical properties of Ca^2+^ currents. However, the effect of Ca_V_β subunits on channel trafficking to and from the plasma membrane is still controversial. Here, we have investigated the impact of Ca_V_β1b and Ca_V_β2a subunits on plasma membrane trafficking of Ca_V_1.2 using a live-labeling strategy. We show that the Ca_V_β1b subunit is more potent in increasing Ca_V_1.2 expression at the plasma membrane than the Ca_V_β2a subunit and that this effect is not related to modification of intracellular trafficking of the channel (i.e. neither forward trafficking, nor recycling, nor endocytosis). We conclude that the differential effect of Ca_V_β subunit subtypes on Ca_V_1.2 surface expression is likely due to their differential ability to protect Ca_V_1.2 from degradation.

## Introduction

Calcium influx generated by voltage-gated calcium channels plays a critical role in neuronal functions such as excitability and gene transcription [1, 2]. High-voltage-activated (HVA) Ca^2+^ channels are formed by assembly of several subunits including the pore forming Ca_V_α_1_ subunit and auxiliary Ca_V_α_2_δ and Ca_V_β subunits [3].

Among HVA channels, Ca_V_1.2 is the most abundant in the mammalian brain [4] and it is localized in clusters in dendritic shafts and spines [5, 6]. Auxiliary Ca_V_β subunits are key modulators of channel biophysical properties and their targeting to the plasma membrane [7, 8]. Four Ca_V_β isoforms have been identified and they are all expressed in the brain. They contain 2 highly conserved domains: a Src Homology 3 domain and a guanylate kinase domain and variable N-terminal, Hook and C-terminal domains. The guanylate kinase domain binds to the α-interacting domain (AID) in the intracellular I–II loop of Ca_V_α_1_ subunits. The binding of Ca_V_β to the AID protects Ca_V_α_1_ from degradation by the proteasome [9]. However, the impact of Ca_V_β subunits on the trafficking of Ca_V_1.2 to and from the plasma membrane is still a matter of debate [10–12].

In this study, we investigated the effects of two Ca_V_β subunits, the cytoplasmic Ca_V_β1b subunit and the membrane anchored (palmitoylated) Ca_V_β2a subunit [13, 14], on plasma membrane trafficking of Ca_V_1.2 using a live-labeling strategy based on a Ca_V_1.2 construct tagged with bungarotoxin binding sites. We show that Ca_V_β1b is more potent in increasing Ca_V_1.2 expression at the plasma membrane than Ca_V_β2a and that this effect is not linked to modification of either forward trafficking, recycling or endocytosis. We suggest that the effect of different Ca_V_β subunits on Ca_V_1.2 surface expression is likely due to their differential ability to protect Ca_V_1.2 from degradation.

## Materials and methods

### Molecular biology

Alpha-bungarotoxin binding sites (BBS: WRYYESSLEPYPD) were inserted between the S5 and the P loop of domain II of Ca_V_1.2 (downstream Q683: FDEMQ-BBS-TRRST) using standard molecular biology techniques. Briefly, two oligonucleotides coding for a BBS and flanked by *Mlu*I restriction sites (oligo A: 5ʹ-ACGCGTCGGACCGGTTGGAGATACTACGAGAGCTCCCTGGAGCCCTACCCTGACCGT A-3ʹ; oligo B: 5ʹ-CGCGTACGGTCAGGGTAGGGCTCCAGGGAGCTCTCGTAGTATCTCCAACCGGTCCGA-3ʹ) were synthesized, annealed and cloned into pMT2 Ca_V_1.2 construct (rat brain Ca_V_1.2 from T. Snutch; GenBank: M67515.1) linearized with *Mlu*I. Correct orientation and location of oligonucleotide cloning were confirmed by sequencing the plasmids. A triple BBS construct was generated.

### Cell culture and transfection

tsA-201 cells were cultured as previously described [15]. Cells were transfected with plasmid encoding rat Ca_V_1.2 (WT or BBS), rat Ca_V_α_2_δ-1 (GenBank: NM_012919.3) and either rat Ca_V_β1b (GenBank: NM_017346.1) or rat Ca_V_β2a (GenBank: NM_053851.1) using the calcium phosphate method. For electrophysiological experiments, cDNA encoding GFP was co-transfected and used as a transfection marker. Apart from dynamin1 K44E [16] which was cloned into pcDNA3.1, all constructs were cloned into the pMT2 vector.

### Electrophysiology recordings

Twenty-four hours after transfection, tsA-201 cells were transferred to a 30 °C incubator for 48 h before being used for experiments. Whole-cell patch-clamp recordings were performed and analyzed as described previously [15]. Briefly, currents were recorded at room temperature (22–24 °C) using an Axopatch 200B amplifier and pClamp 9.2 software. Patch pipettes were filled with a solution containing the following (in mM): 130 CsCl, 2.5 MgCl_2_, 10 HEPES, 5 EGTA, 3 Na-ATP, 0.5 Mg-GTP, pH 7.4. The external solution contained the following (in mM): 132.5 CsCl, 1 MgCl_2_, 10 HEPES, 5 BaCl_2_, 10 glucose, pH 7.4. Current–voltage relationships were obtained by applying 250 ms pulses ranged from − 50 to + 50 mV in 5 mV increment from a holding potential of − 100 mV. Current density–voltage relationships were fitted with a modified Boltzmann equation as follows: I = (G_max_ × (V − V_rev_))/(1 + exp(−(V − V_50,act_)/k)), where I is the current density (in pA/pF), G_max_ is the maximum conductance (in nS/pF), V_rev_ is the reversal potential, V_50,act_ is the midpoint voltage for current activation and k is the slope factor.

### Trafficking assays and confocal microscopy

tsA-201 cells were plated onto glass-bottomed dishes (MatTek Corp., Ashland, MA) precoated with poly-l-lysine and transfected as described above. After 3 days expression, cells were washed twice with Krebs–Ringer solution with HEPES (KRH) (in mM; 125 NaCl, 5 KCl, 1.1 MgCl_2_, 1.2 KH_2_PO_4_, 2 CaCl_2_, 6 Glucose, 25 HEPES, 1 NaHCO_3_). For endocytosis experiments, cells were incubated with 10 µg/ml α-bungarotoxin Alexa Fluor^®^ 488 conjugate (BTX488) (Thermo Fisher Scientific) at 17 °C for 30 min. The unbound BTX488 was removed by washing with KRH, and the labelled cells were returned to 37 °C. Endocytosis was terminated by fixing the cells with cold 4% PFA in PBS for 5 min, and then permeabilized with 0.05% Triton X-100 in PBS for 10 min. Cells were blocked with 10% FBS in PBS for at least 30 min and incubated with the primary Ab (rabbit anti-Ca_V_1.2, 1:200, Alomone labs) for 1 h at room temperature. Samples were washed and incubated with secondary conjugated Ab anti-rabbit AF594 (1:500; Thermo Fisher Scientific) for 1 h at room temperature. After washing, samples were covered with SlowFadeTM Gold antifade mountant (Thermo Fisher Scientific). For the forward trafficking assay, the cells were incubated with 10 μg/ml unlabeled α-bungarotoxin (BTX; Thermo Fisher Scientific) at 17 °C for 30 min. The unbound BTX was washed off with KRH, and the cells were then incubated with 10 μg/ml BTX488 in KRH at 37 °C. To stop the reaction, cells were washed twice with cold KRH and then fixed with 4% PFA in PBS. Brefeldin A (BFA; 200 ng/ml (0.71 μM); Sigma-Aldrich) in 0.4% DMSO was added to the cells in FBS-free culture medium for 4 h before the experiment, and during the experiment in KRH buffer. Cells were examined on a Leica SP8 confocal microscope using a 63×/1.4 numerical aperture oil-immersion objective in 16-bit mode. Acquisition settings, chosen to ensure that images were not saturated, were kept constant for each experiment.

### Statistical analysis

Data are given as mean ± SEM. Statistical comparisons were performed using paired and unpaired Student’s t tests, as appropriate, using SigmaPlot 14.5 or Prism GraphPad. Differences were considered to reach statistical significance when p < 0.05.

## Results and discussion

**Fig. 1:**
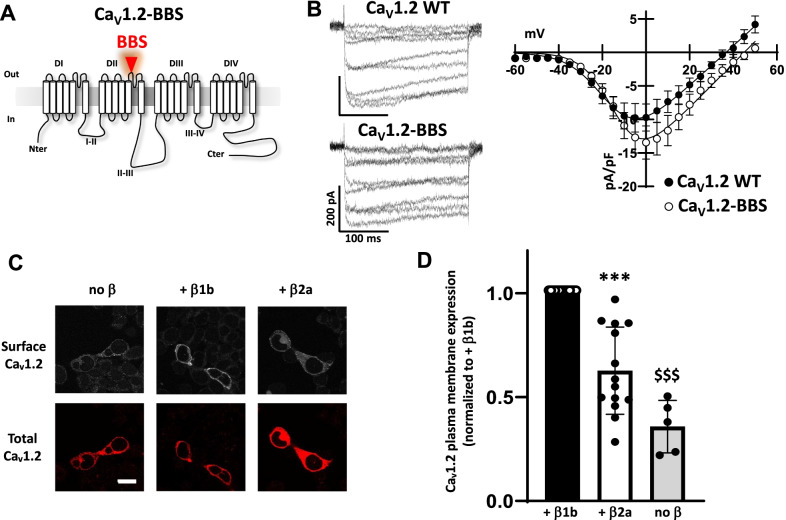
Effect of Ca_V_β subunits on Ca_V_1.2 cell surface expression in tsA-201 cells. **a** Schematic of Ca_V_1.2 channel tagged with bungarotoxin binding site (Ca_V_1.2-BBS, BBS: red triangle) between S5 and the P-loop of domain II (DII). **b** Representative whole-cell current traces recorded in response to depolarizing steps from − 50 to + 40 mV from a holding potential of − 100 mV from tsA-201 cells expressing either Ca_V_1.2 WT (top traces) or Ca_V_1.2-BBS (bottom traces) together with auxiliary subunits Ca_V_α_2_δ-1 and Ca_V_β1b. Mean I/V curves (right panel) for Ca_V_1.2 WT (filled circle, n = 9) and Ca_V_1.2-BBS (open circle, n = 18) co-expressed with auxiliary subunits Ca_V_α_2_δ-1 and Ca_V_β1b. **c** Confocal images showing plasma membrane expression of Ca_V_1.2-BBS in tsA cells stained with α-bungarotoxin (BTX)-AF488 (top panels). Ca_V_1.2-BBS was co-expressed with Ca_V_α_2_δ-1 (left, no β) and either Ca_V_β1b (center) or Ca_V_β2a (right). Cells were incubated at 17 °C with BTX-AF488 for 30 min and fixed. The cells were then permeabilized and stained with a rabbit anti-Ca_V_1.2 Ab and secondary Ab anti-rabbit AF594 (bottom panels). Scale 20 µm. **d** Average Ca_V_1.2-BBS surface expression co-transfected with Ca_V_α_2_δ-1 and either Ca_V_β1b (black bar), or Ca_V_β2a (open bar), or empty vector (gray bar). Bars are mean (± SEM) normalized to Ca_V_β1b mean. ***p < 0.001, n = 14; ^$$$^*p* < 0.001, n = 5; paired t-test, n numbers correspond to independent experiments

To monitor the trafficking of Ca_V_1.2 to the plasma membrane we introduced α-bungarotoxin binding sites in the extracellular loop of the channel (Fig. 1a). This construct, Ca_V_1.2-BBS, remained functional and generated Ba^2+^ currents with a density similar to the WT channel (− 9.7 ± 2.0 pA/pF, n = 9, vs − 13.5 ± 2.5 pA/pF, n = 18, for Ca_V_1.2-BBS and WT, respectively; Fig. 1b). However, the insertion of the tag induced a slight increase in the slope of the activation curve (from − 9.3 ± 0.9 mV, n = 9, to − 7.1 ± 0.3 mV, n = 18, for WT and Ca_V_1.2-BBS, respectively, *p* = 0.007) and a depolarizing shift of the reversal potential (from 37.5 ± 2.6 mV, n = 9, to 48.4 ± 2.4 mV, n = 18, for WT and Ca_V_1.2-BBS, respectively, *p* = 0.01). The V_50,act_ and the G_max_ remained unchanged (V_50,act_ = − 14.5 ± 2.2 mV, n = 9, and − 14.7 ± 0.6 mV, n = 18; G_max_ = 0.34 ± 0.08 nS/pF, n = 9, and 0.30 ± 0.05 nS/pF, n = 18, for WT and Ca_V_1.2-BBS, respectively). The effect on reversal potential may be indicative of an effect of the BBS modification on permeability, but this should have little bearing on the utility of this construct for trafficking studies.

We subsequently checked the cell surface expression of Ca_V_1.2-BBS and compared the effect of co-expressing different types of auxiliary Ca_V_β subunits. Three days after transfection, tsA-201 cells were live-labelled with α-BTX-488 for 30 min at 17 °C, fixed and the fluorescence was quantified (Fig. 1c and d). We found that the co-expression of Ca_V_β1b induced a 60% increase in Ca_V_1.2-BBS surface expression compared with no Ca_V_β. Additionally, although the co-expression of Ca_V_β2a also increased Ca_V_1.2-BBS cell surface expression, its effect was not as marked as Ca_V_β1b since only a 30% increase of fluorescence was recorded (Fig. 1c and d). This result is in good agreement with a study showing differential effects of Ca_V_β subunits on Ca_V_1.2-generated current densities, although it is important to note that interpretations of electrophysiological measurements can be confounded by effects on channel biophysics [17].

**Fig. 2:**
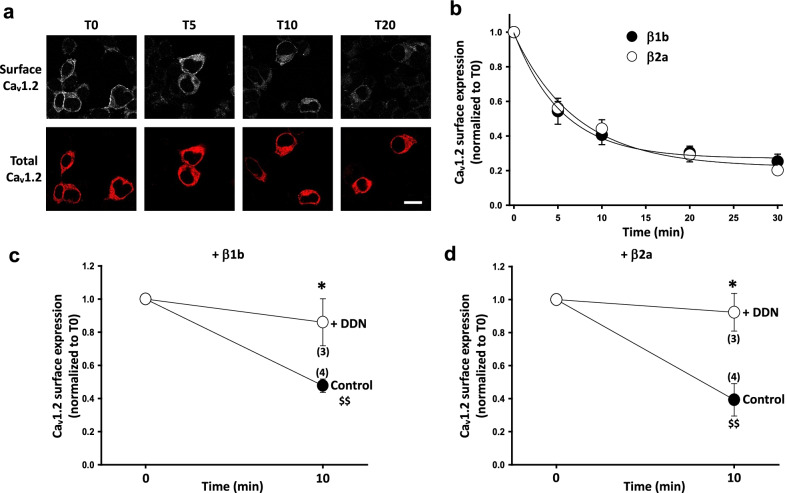
Ca_V_1.2 endocytosis is dynamin-dependent and Ca_V_β subtype-independent. **a** Representative confocal images of tsA-201 cells expressing Ca_V_1.2-BBS and labelled with BTX-AF488 (top panels). Ca_V_1.2-BBS was co-expressed with Ca_V_α_2_δ-1 and Ca_V_β1b. Cells were incubated at 17 °C with BTX-AF488 for 30 min and then fixed at different time point after incubation at 37 °C, from zero (T0) to 20 min (T20). The cells were then permeabilized and stained with a rabbit anti-Ca_V_1.2 Ab and a secondary Ab anti-rabbit AF594 (bottom panels). Scale bar 20 µm. **b** Time course of endocytosis of cell surface Ca_V_1.2-BBS co-expressed with Ca_V_α_2_δ-1 and either Ca_V_β1b (filled circle) or Ca_V_β2a (open circle). The results are shown as the mean ± SEM. The n numbers correspond to independent experiments (average fluorescence from at least 25 cells per time point). The data were fitted with single exponentials. The time constants of the fits were 5.6 ± 0.2 min for Ca_V_β1b and 7.1 ± 0.2 min for Ca_V_β2a, respectively. **c** and **d** Effect of dominant negative dynamin Dyn K44E on Ca_V_1.2 endocytosis. Cells were transfected with Ca_V_1.2-BBS, Ca_V_α_2_δ-1 and either Ca_V_β1b (**c**) or Ca_V_β2a (**d**) together with either empty pcDNA3.1 vector (filled symbols) or Dyn K44E (DDN, open symbols). Cells were incubated at 17 °C with BTX-AF488 for 30 min and then fixed at time point T0 and T20 after incubation at 37 °C. Cells were subjected to immunocytochemistry as described in **a**. BTX-AF488 fluorescence was normalized to the mean fluorescence at T0 for each condition. The results are shown as the mean ± SEM. The n numbers correspond to independent experiments (average fluorescence from at least 25 cells per time point). ^$$^*p* < 0.01 Control T10 vs Control T0; *p < 0.05 DDN T10 vs Control T10, unpaired t-test

We then aimed to gain insight into the Ca_V_β subunit-dependent mechanism(s) responsible for the effects on Ca_V_1.2 surface expression. Plasma membrane expression of Ca_V_1.2 results from the balance between the incorporation of newly synthetized Ca_V_1.2 from the ER, recycled Ca_V_1.2 from endosomal compartments and the removal of channels from the plasma membrane by endocytosis [18]. We first monitored the impact of Ca_V_β subunits on Ca_V_1.2 endocytosis by comparing the rate of internalization of Ca_V_1.2-BBS (Fig. 2). We showed that Ca_V_1.2-BBS, either co-expressed with Ca_V_β1b or Ca_V_β2a, exhibited similar kinetics of endocytosis with a time constant of ~ 6 min (Fig. 2b). This is in line with previous studies on N-type calcium channels [19, 20] and measurements on cardiac cell lines [21]. We note that when Ca_V_β subunits are not co-expressed, no reduction of Ca_V_1.2-BBS fluorescence is detected over the duration of BTX incubation (after 20 min, Ca_V_1.2-BBS fluorescence represented 100 ± 12% of the initial fluorescence, n = 3), suggesting that Ca_V_β subunits promote Ca_V_1.2 endocytosis. This conclusion is different from that of a recent study showing that stabilizing the Ca_V_1.2–Ca_V_β2a interaction via the creation of a concatemer increases the retention time of Ca_V_1.2 at the plasma membrane in HEK-293 and HLA-1 cells [12]. However, in our experimental conditions, the starting level of Ca_V_1.2-BBS fluorescence without Ca_V_β subunit is very low, close to the detection limit, and we cannot exclude that some endocytosis of channels may take place even in the absence of Ca_V_β. It was previously shown that Ca_V_1.2 internalization is dynamin-dependent [21]. We took advantage of the dominant negative effect of the dynamin mutant K44E [16] to show that Ca_V_1.2 internalization depends on dynamin, regardless of co-expressed Ca_V_β subtype (Fig. 2c and d).

**Fig. 3:**
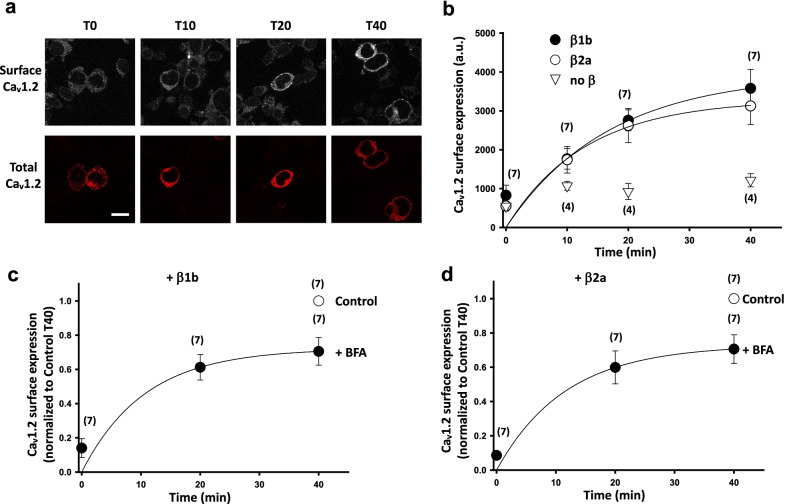
Ca_V_1.2 forward trafficking and recycling are not Ca_V_β-subtype dependent. **a** Confocal images of tsA-201 cells expressing Ca_V_1.2-BBS and labelled with BTX-AF488 (top panels). Ca_V_1.2-BBS was co-expressed with Ca_V_α_2_δ-1 and Ca_V_β1b. Cells were incubated at 17 °C with untagged BTX for 30 min and then incubated at 37 °C with BTX-AF488. The cells were fixed at different time point after incubation at 37 °C, from zero (T0) to 40 min (T40). The cells were then permeabilized and stained with a rabbit anti-Ca_V_1.2 Ab and a secondary Ab anti-rabbit AF594 (bottom panels). Scale bar 20 µm. **b** Time course of insertion of Ca_V_1.2-BBS at the cell surface when co-expressed with Ca_V_α_2_δ-1 and either Ca_V_β1b (filled circle), Ca_V_β2a (open circle) or empty vector (open triangle). The results are shown as the mean ± SEM (n numbers correspond to independent experiments). Data were fitted with single exponentials. The time constants of the fits were 16.8 ± 12.6 min and 13.0 ± 14.2 min for Ca_V_β1b and Ca_V_β2a (n = 7), respectively. **c** and **d** Effect of Brefeldin A (BFA) treatment on Ca_V_1.2 forward trafficking. tsA-201 cells were co-transfected with Ca_V_1.2-BBS, Ca_V_α_2_δ-1 and either Ca_V_β1b (**c**) or Ca_V_β2a (**d**). Cells were treated with BFA for 4 h before undergoing the forward trafficking protocol described in **a**. BTX-AF488 fluorescence was normalized to the mean fluorescence at T40 for the control condition (open circle). The results are shown as the mean ± SEM. The n numbers correspond to independent experiments (average fluorescence from at least 25 cells per time point). The data were compared using an unpaired t-test. The data were fitted with single exponentials. The time constants of the fits were 10.6 ± 6.6 min and 11.6 ± 8.1 min for Ca_V_β1b and Ca_V_β2a (n = 7), respectively

Next, we examined the effect of Ca_V_β subunits on net forward trafficking by monitoring the insertion of Ca_V_1.2-BBS into the plasma membrane as a function of time (Fig. 3a and b). In the no Ca_V_β condition, Ca_V_1.2-BBS surface expression doubled after 10 min and stayed stable during the next 40 min. However, when Ca_V_β subunits were co-expressed, we recorded an increase of Ca_V_1.2-BBS surface expression that reached a plateau after 40 min. The increases were comparable for both Ca_V_β subunits and represented ~ 5 times the starting amount of Ca_V_1.2-BBS surface expression. This is surprising, given that the steady state level of Ca_V_1.2 is higher in the presence of the Ca_V_β1b isoform (see below).

Finally, we used BFA to disrupt the Golgi apparatus and prevent the transfer of newly synthesized channels from the ER to the plasma membrane (Fig. 3c and d). Using this strategy, we were able to estimate that 70% of the surface Ca_V_1.2-BBS originated from a recycling pathway [21] and we could also attribute 30% to a forward trafficking process. These contributions remained identical irrespective of whether Ca_V_1.2-BBS was co-expressed with Ca_V_β1b or Ca_V_β2a subunits. Interestingly, the contributions recycling/forward trafficking for Ca_V_2.2 cell surface expression were reported to be closer to 50% [19, 20], although it is important to highlight the fact that these studies were performed in a neuronal cell line and that further investigations would be needed to rule out a cell-dependent effect.

Altogether, we showed that the Ca_V_β subunit subtype dependent effect on Ca_V_1.2 surface expression was not associated with any modifications of the kinetics for forward trafficking, endocytosis and recycling. These results suggest that the level of Ca_V_1.2 (available in the ER to be trafficked to the plasma membrane) is differentially increased in the presence of different Ca_V_β subunits. Such a mechanism is supported by the conclusions of a previous study from our group that showed that Ca_V_β subunits protect Ca_V_1.2 from ubiquitination and degradation by the proteasome [9]. If so, then the fact that the observed effects were greater with Ca_V_β1b than with Ca_V_β2a suggests the possibility that a difference in the amount of Ca_V_1.2 in the ER could be due to a differential ability of the two Ca_V_β subunits to protect Ca_V_1.2 from degradation. This could potentially be due to the selective palmitoylation and membrane anchoring of Ca_V_β2a compared to the pure cytoplasmic expression of Ca_V_β1b which may have better access to the pore forming subunit in the ER. Alternatively, it is possible that the latter may be expressed at higher levels than the former and thus more effective in protecting the channel from degradation. We also consider the possibility that the protective effects of the Ca_V_β subunit may not be dependent on a physical interaction with the Ca_V_1.2, but instead act by regulating calcium channel expression at the transcriptional or translational level. For example, for Ca_V_3 calcium channels, coexpression of ancillary subunits promotes current densities despite an absence of a physical interaction [22]. On the other hand, mutation of Ca_V_1.2 residue W440 which prevents the physical association with the Ca_V_β subunit leads to compromised membrane expression of the channel [9, 23], thus arguing against a diffuse effect on Ca_V_1.2 protein expression. Overall, our data are consistent with a mechanism by which Ca_V_β subunits are more important for regulating the levels of Ca_V_1.2 channels at the level of the ER, rather than directly altering the forward and reverse plasma membrane trafficking of the channel complexes. This does not negate the possibility that these subunits may be involved in modulating the targeting of Ca_V_1.2 channels to specific sub-loci within the plasma membrane.

## Data Availability

Data and materials will be made available based on reasonable request.
